# Ultrastructural characteristics of erythroid cells in congenital dyserythropoietic anemia type II, with a focus on peripheral cisternae and double membranes

**DOI:** 10.1097/BS9.0000000000000136

**Published:** 2022-10-10

**Authors:** Yong-Xin Ru, Shu-Xu Dong, Jing Liu, Brian Eyden

**Affiliations:** a State Key Laboratory of Experimental Hematology, National Clinical Research Center for Blood Diseases, Haihe Laboratory of the Cell Ecosystem, Institute of Hematology and Blood Diseases Hospital, Chinese Academy of Medical Sciences and Peking Union Medical College, Tianjin, China;; b Formerly: Department of Histopathology, Christie Hospital NHS Trust, Manchester, UK

**Keywords:** Congenital dyserythropoietic anemia type II, Erythroid cells, Peripheral cisternae, Ultrastructural

## Abstract

Peripheral cisternae and double membranes (PCDMs) in erythroid cells are a landmark of type II congenital dyserythropoietic anemia (CDA). To gain further insights into the mechanism of dyserythropoiesis, erythroblasts and erythrocytes in bone marrow were studied in 22 Chinese patients with CDA Ⅱ by transmission electron microscopy. The study demonstrated an increase in all patients in erythroblasts with PCDMs with development from pro-erythroblast to red blood cells. PCDMs often connected with cisternae of endoplasmic reticulum (ER) and the perinuclear space, and were accompanied by karyopyknosis, karyolysis and disruption in polychromatic and orthochromatic erythroblasts. The results suggest that PCDMs are transformed from ER during erythropoiesis and participate in the dissolution and deletion of late erythroid cells in patients with CDA II.

## 1. INTRODUCTION

Congenital dyserythropoietic anemia (CDA) are a heterogenous group of rare inherited diseases characterized by ineffective erythropoiesis. The dysplastic changes affect erythroblasts from early to late stages, leading to dyserythropoiesis with structural alterations and disruption of erythroblasts in the bone marrow.^1^ CDA II is the most common form, followed by CDA I, the prevalence being about 0.71 and 0.24 cases/million in Europe respectively.^2^ The incidence rate of CDAs has not been summarized in Asia although cases of CDA I and II from China have been reported frequently. Chinese patients usually suffer from moderate anemia (hemoglobin from 5.5 to 12.2 g/dL), chronic hemolysis, splenomegaly, and jaundice, and in some cases, iron overload due to multiple blood transfusions.^3,4^ Defining morphologic features of CDA II include erythroid hyperplasia, binuclearity, and chromatin bridging in erythroblasts, a positive acidified serum lysis test (Ham test) and an autosomal recessive character due to bi-allelic mutations in the SEC23B gene (20p11.23).^5–7^

Peripheral cisternae and discontinuous double membranes (PCDMs) are a distinctive feature of erythroid cells in CDA II as seen by transmission electron microscopy (TEM). PCDMs were identified by the three proteins—GRP78, calreticulin and PDI—originally found on normal endoplasmic reticulum (ER) and tested for using the under-glycosylation of erythrocyte membrane band 3 (SDS-PAGE test); this revealed that PCDMs originated from residual ER in CDA II.^8,9^

To advance our understanding of PCDMs and their significance in erythroblasts, the present study reports the results of observations on ultrastructural alterations of erythroid cells at different stages in 22 cases of CDA II from the Blood Diseases Hospital, Tianjin.

## 2. MATERIALS AND METHODS

### 2.1. Clinical details

Twenty-two cases of CDA II were diagnosed based on: (1) clinical presentations of moderate anemia, jaundice and splenomegaly; (2) morphologic features of erythroblastic hyperplasia, binuclearity of erythroblasts on light microscopy and PCDMs on TEM; (3) laboratory examinations of positive Ham test and SDS-PAGE test. Twelve cases had a mutation in the SEC23B gene (20p11.23) as reported previously.^3,4,10^

### 2.2. Transmission electron microscopy

Mononuclear cells and bone-marrow granules were isolated from bone-marrow aspirates and processed according to standard TEM procedures. Briefly, the samples were fixed in 2.5% glutaraldehyde, postfixed in 1% osmium tetroxide, washed in phosphate-buffered saline, dehydrated in graded alcohols and embedded in Epon 812. Ultrathin sections at 60nm were cut and stained with uranyl acetate and lead citrate. Erythroid cells were observed by TEM.

## 3. RESULTS

**Figure 1.**
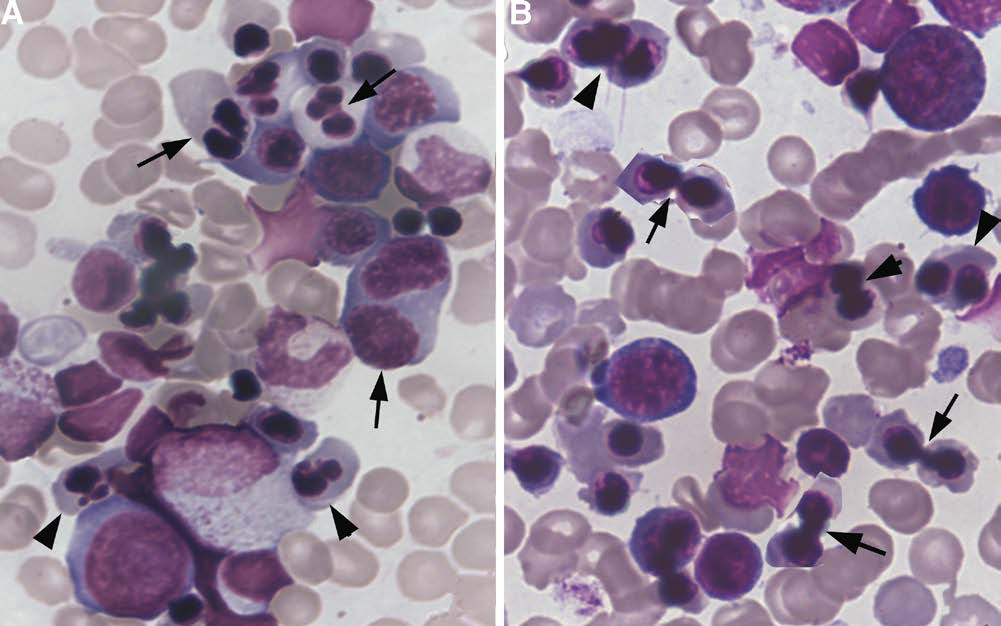
Morphologic feature of erythrocytes in CDA Ⅱ. (A) erythroid hyperplasia, binuclearity (arrow) and erythroblast karyorrhexis (arrowheads), ×1 K; and (A) chromatin bridging erythroblasts (arrows) and karyopyknosis (arrowheads) ×1 K. CDA = congenital dyserythropoietic anemia.

Morphologic features as seen in bone-marrow smears were characterized by erythroid hyperplasia, binuclearity, chromatin bridging, karyorrhexis, karyopyknosis and disruption of erythroblasts (Fig. 1).

### 3.1. Characteristics of proerythroblasts

**Figure 2.**
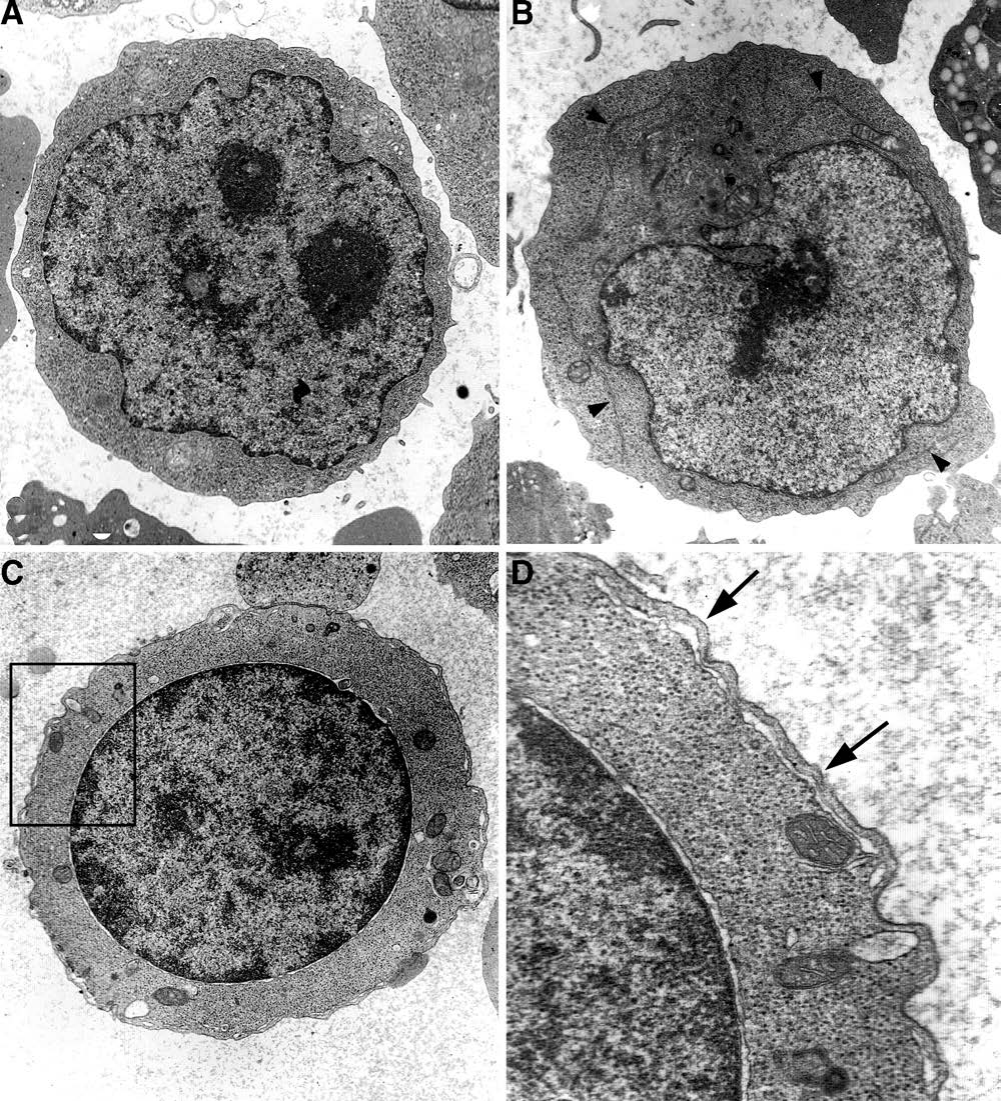
Ultrastructural characteristics of erythroblasts. (A) pro-erythroblast shows normal features, including a nucleus and prominent nucleoli, ×5K; (B) a pro-erythroblast contains long profiles of ER in the cytoplasm (arrowheads), ×5K; (C) a basophilic erythroblast has PCDM, ×5K; (D) from (C) shows a peripheral cytoplasmic band (arrows) and a cisterna close to the surface, ×20K. ER = endoplasmic reticulum; PCDM = peripheral cisternae and double membrane.

Most proerythroblasts had a normal size and morphology, showing a round nucleus, prominent nucleolus, and long cisternae of ER, lysosomes, and mitochondria in the cytoplasm, but a few of the cells showed PCDMs in the peripheral cytoplasm (Fig. 2A and B).

### 3.2. Characteristics of basophilic erythroblasts

**Figure 3.**
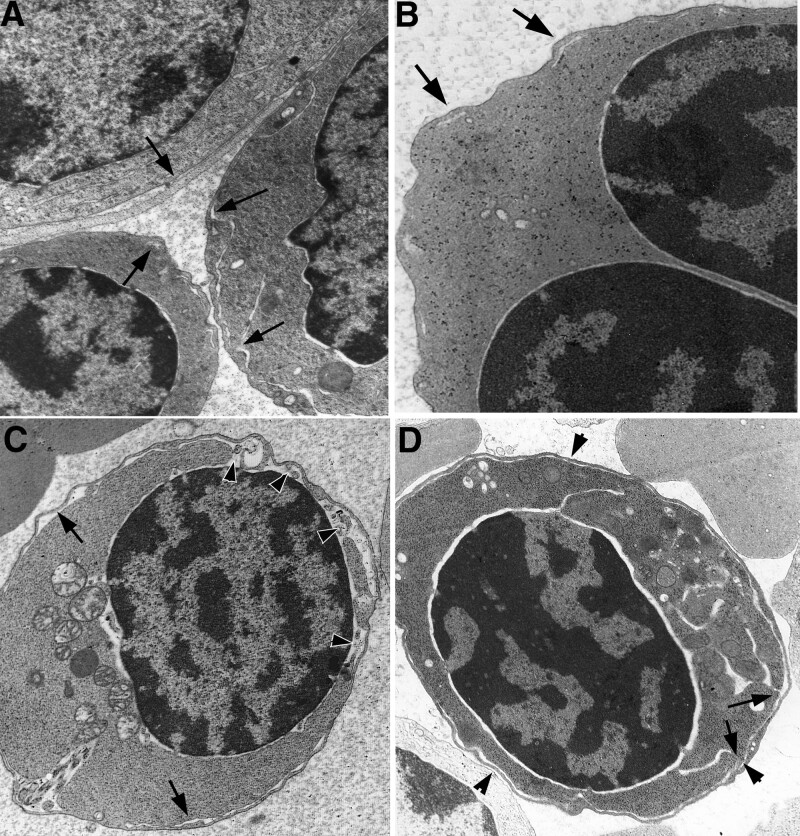
Ultrastructural characteristics of erythroblasts. (A) A regular PCDM at the surface of a pro-erythroblast (upper left) and uneven PCDM (arrows) on basophilic erythroblasts (lower), ×8K; (B) a discontinuous PCDM on a binucleated erythroblast, ×10K; (C) a peripheral cisterna is expanded and connected with the perinuclear space (arrowheads) in a polychromatic erythroblast, ×5K; (D) a peripheral cisterna connected with the ER (arrows) in a polychromatic erythroblast, ×5K. PCDM = peripheral cisternae and double membrane.

Basophilic erythroblasts usually had a normal appearance of the nucleus and cytoplasmic organelles. Some basophilic erythroblasts contained PCDMs and proportions varied in different cases. The peripheral cisternae of PCDMs were usually narrow and discontinuous. Peripheral cytoplasmic bands separated by the cisternae were often thin close to the cell surfaces (Figs. 2C and D and 3A).

### 3.3. Characteristics of polychromatic erythroblasts

**Figure 4.**
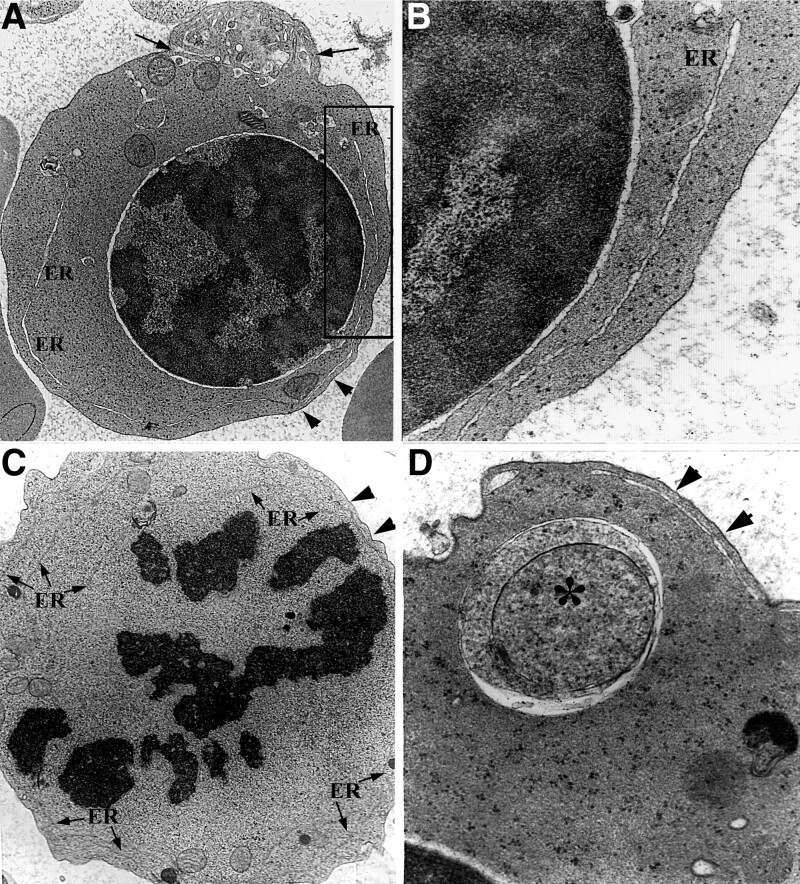
Ultrastructural characteristics of polychromatic erythroblasts. (A) An orthochromatic erythroblast has a cluster of PCDM (arrows), and a profile of PCDM (arrowheads) continuous with ER in the deep cytoplasm, ×5K; (B) from (A), PCDM continuous with ER, ×15K; (C) a mitotic erythroblast has a PCDM (arrowheads) at the surface along with ER in the cytoplasm (arrows), ×5K; (D) an autophagosome (asterisks) in an erythroblast with PCDM, ×10K. ER = endoplasmic reticulum; PCDM = peripheral cisternae and double membrane.

**Figure 5.**
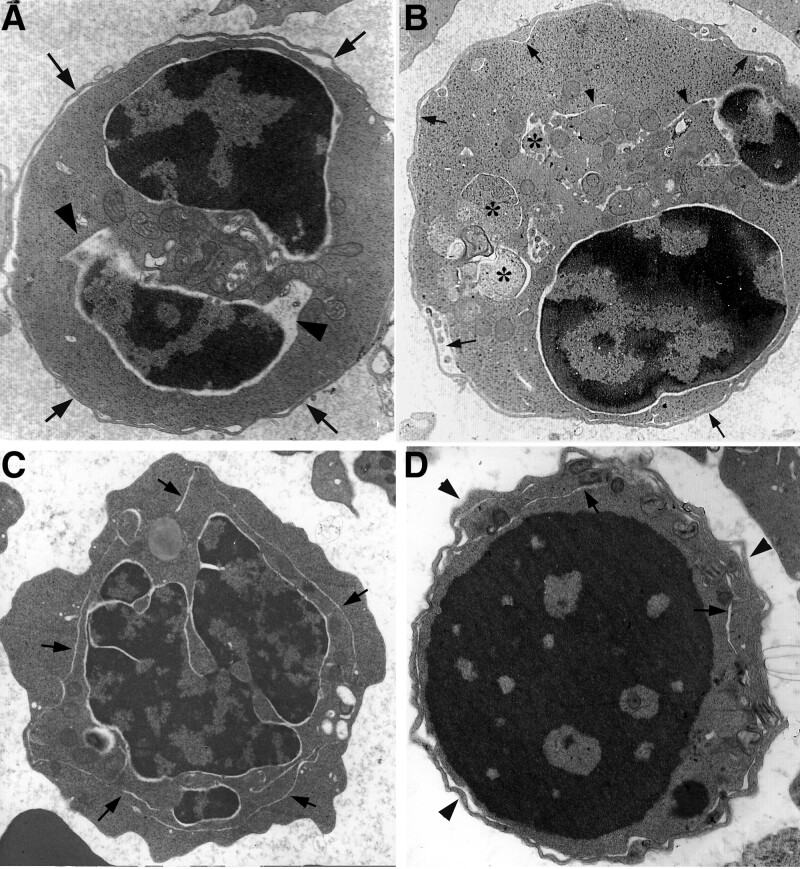
Ultrastructural characteristics of polychromatic erythroblasts. (A) A binucleated erythroblast with PCDM (arrows) and an expanded perinuclear space (arrowheads), ×5K; (B) a binucleated erythroblast with PCDM contains mitophagosomes (asterisks) and ER (arrows), ×6K; (C) an erythroblast contains a bizarre nucleus and ER (arrows), ×5K; and (D) PCDM (arrowheads) is connected with ER (arrows) in a karyopyknotic erythroblast, ×8K. ER = endoplasmic reticulum; PCDM = peripheral cisternae and double membrane.

Polychromatic erythroblasts in patients with CDA II were mostly characterized by a PCDM in which peripheral cisternae were often expanded and connected with cisternae of ER and perinuclear spaces. The ER connected with PCDMs was often characterized by degenerated features such as an electron-dense appearance and a lack of ribosomes (Fig. 3B–D). Peripheral cytoplasmic bands separated by PCDMs were uneven in width, some thick and others thin (Fig. 3A). PCDMs of some polychromatic erythroblasts ran into the deep cytoplasm and connected with narrow profiles of ER (Fig. 4**A and B**). Some mitotic polychromatic erythroblasts also showed PCDMs connecting with ER (Fig. 4C). Autophagosomes were often found in polychromatic erythroblasts with PCDMs (Figs. 4D and 5B).

### 3.4. Characteristics of orthochromatic erythroblasts

**Figure 6.**
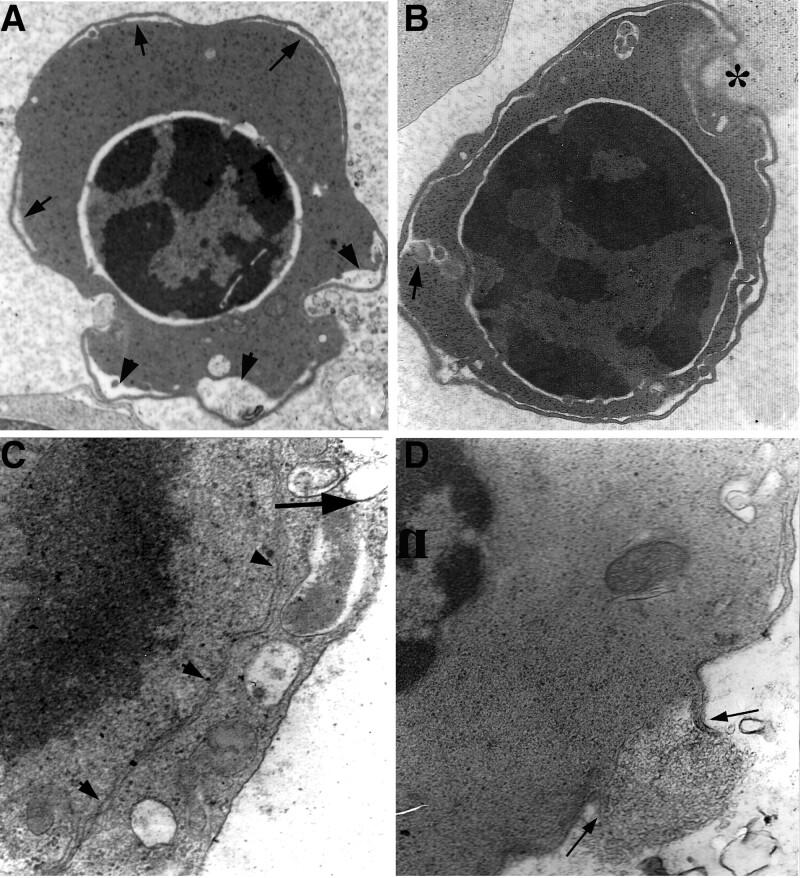
Ultrastructural characteristics of orthochromatic erythroblasts. (A) PCDM of an orthochromatic erythroblast includes narrow areas (arrows) and wider areas of cisternae (arrowheads), ×5K; (B) PCDM stretches into the cytoplasm (arrow) and shows features of damage (asterisk), ×6K; (C) PCDM parallel with ER (arrowheads) and is broken (arrow) on the surface of a karyolytic erythroblast, ×15K; and (D) a broken PCDM (arrows) of an erythroblast, ×15K. ER = endoplasmic reticulum; PCDM = peripheral cisternae and double membrane.

Orthochromatic erythroblasts in CDA Ⅱ usually had a nucleus with condensed chromatin and a higher density of cytoplasm than normal. However, some of them were characterized by binuclearity, bizarre nuclei, karyorrhexis, karyopyknosis, and PCDMs. Some orthochromatic erythroblasts contained long ER cisternae and cytoplasmic autophagosomes in the cytoplasm (Fig. 5B and C). Interestingly, PCDM of the orthochromatic erythroblasts stretched into deep cytoplasm, and showed expansion of cisternae and broken features (Fig. 5D and Fig. 6).

### 3.5. Ultrastructural characteristics of erythrocytes

**Figure 7.**
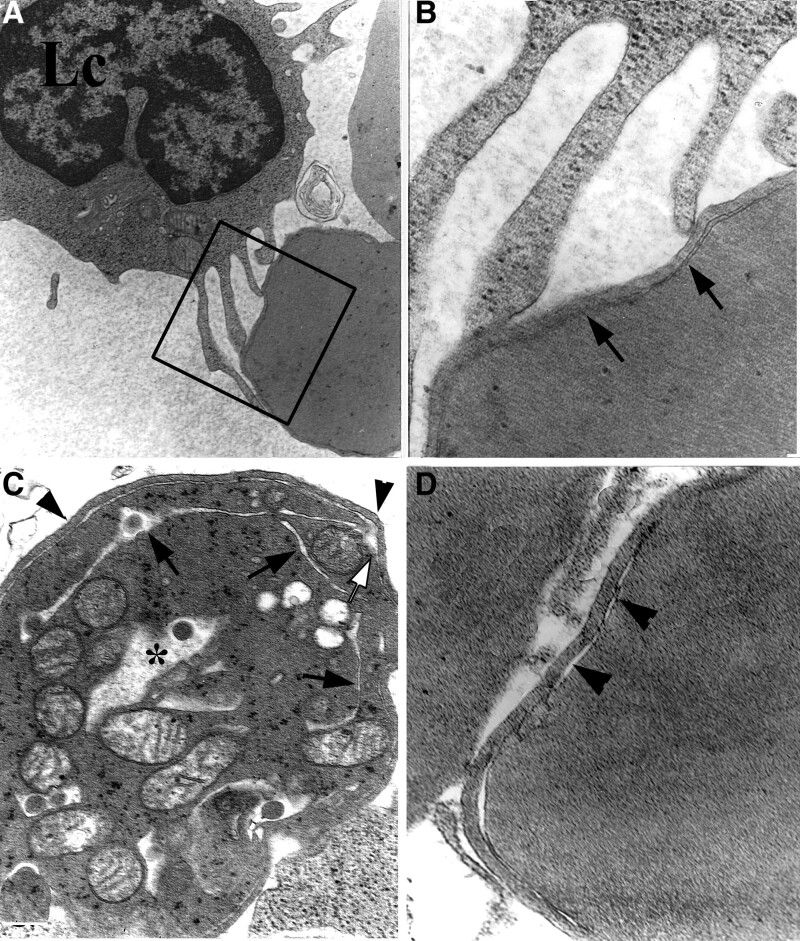
Ultrastructural characteristics of erythrocytes. (A) An activated lymphocyte (Lc) attaches to an RBC with PCDM, ×5K; (B) from (A) shows a narrow PCDM (arrows), ×15K; (C) PCDM (white arrow) connects with ER (arrows) in a reticulocyte, ×15K; (D) PCDM (arrowheads) of a red blood cell, ×20K. ER = endoplasmic reticulum; PCDM = peripheral cisternae and double membrane; RBC = red blood cell.

A small proportion of reticulocytes and red blood cells (RBCs) showed PCDMs in all of the patients, apart from other erythrocytes. Reticulocytes often included a few dilated mitochondria and degenerated ER profiles connected with PCDM near the surface. Some cisternae of the PCDMs reached into the deeper cytoplasm from points near the surface. (Fig. 7).

## 4. DISCUSSION

CDAs are a group of heterozygous disorders characterized by ineffective erythropoiesis with inadequate reticulocyte values, ineffective erythropoiesis and hemolysis.^11^ All of the patients suffer from moderate anemia, jaundice, and splenomegaly. Older patients with CDAs often present with complications of cholelithiasis and iron overload following frequent blood transfusions.

Striking dysplastic changes in erythroblasts included aberrant erythroblasts in bone marrow and destruction of RBCs in the peripheral circulation.^5,12^ CDA types I, II, and III were originally defined according to distinctive morphologic features of erythroblasts in bone marrow in the 1960s,^13^ although the three types of CDA present with a common process of ineffective erythropoiesis and peripheral hemolysis simultaneously. Specifically, CDA II was characterized by a high percentage of binuclear cells, chromatin bridging on light microscopy, PCDMs in erythroblasts on TEM, and a positive Ham test and SDS-PAGE test in the laboratory.^14^

In CDA II, erythroid cells were predominantly disrupted and decreased as N-glycans released from band 3 were truncated, but immature high mannose and hybrid glycans were increased. These changes were tested by a narrower band size and increasing migration of RBC plasma membrane proteins band 3 and band 4.5 using SDS-PAGE.^15,16^ The details of the mechanism of development of CDA II remain to be clarified in patients given the lack of an analogous animal model although it is an autosomal recessive disease associated with mutations in SEC23B responsible for coding a core component of the coat protein complex.^6^

**Figure 8.**
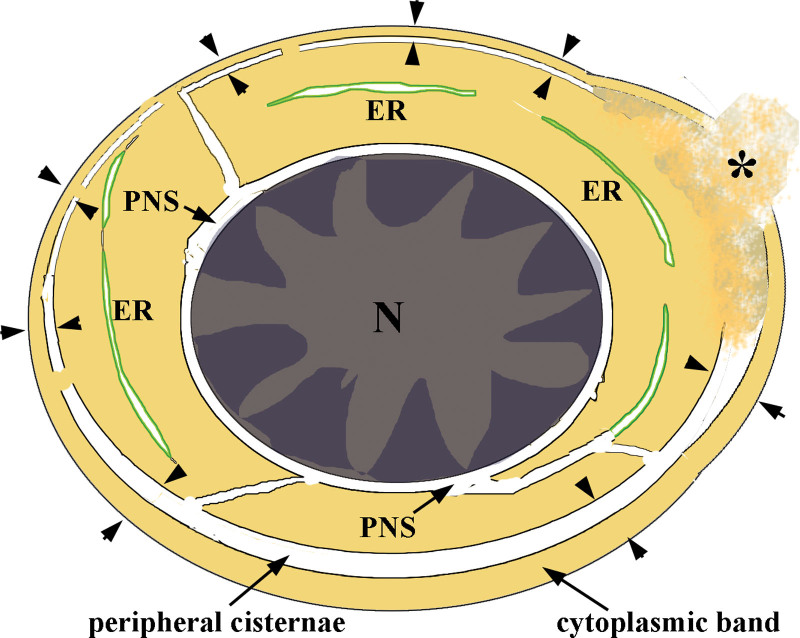
Diagram of aberrant erythroblasts in CDA Ⅱ: the pairs of apposed arrowheads indicate complexes of PCDM and cytoplasmic band; expansion of peripheral cisternae and thickness of cytoplasmic bands varied in erythrocytes at different stages; the peripheral cisterna communicated with ER and the PNS; the asterisk (*) shows damage to the cytoplasmic band at the surface. CDA = congenital dyserythropoietic anemia; ER = endoplasmic reticulum; PCDM = peripheral cisternae and double membrane; PNS = perinuclear space.

As regards PCDMs seen by TEM, these are complexes including three components: a cisterna, discontinuous double membranes and a sheet-like cytoplasmic band (Fig. 8). It is a structural marker of CDA II distinguishing it from other types of CDA, together with erythroid hyperplasia, binuclear and multinuclear erythroblasts, and chromatin bridging as seen morphologically.^17^ The peripheral cisternae of PCDMs often connect with cisternae of ER and the perinuclear space, and discontinuous double membranes also connect with membranes of ER as shown in this study. The phenomenon as seen here is consistent with previous studies, substantiating the idea that PCDMs originate from ER in erythroid cells in CDA II.^8^ The thickness of peripheral cytoplasm separated by the peripheral cisternae was different in erythrocytes, that is, some PCDMs were observed close to the surface, while some PCDMs were located further away from it. The latter peripheral cisternae were often parallel with ER in the deep cytoplasm of erythroblasts and erythrocytes, which provided evidence again of PCDM transformation from ER.

In this study of 22 cases, PCDMs were mostly found in orthochromatic and polychromatic erythroblasts, often in basophilic erythroblasts, and occasionally in proerythroblasts. This indicated that the occurrence of PCDMs was associated with the maturation and development of erythroid cells. Some erythroblasts with PCDMs included a bizarre nucleus and mitotic appearance, and some reticulocytes and RBCs also carried PCDMs. Furthermore, the cisternae of PCDMs were more expanded in late rather than earlier stages of erythroblasts. This indicated that PCDMs were capable of affecting erythroblast proliferation and transferring to the next generation during erythropoiesis.

PCDMs were not only a distinct marker but also played an important role in dyserythropoiesis; however, it has not been clarified in CDA II. The present study demonstrated that PCDMs were dissolved and ruptured at some points on the cell surface of orthochromatic and polychromatic erythroblasts. The erythroblasts showed simultaneous karyopyknosis, karyolysis and cytolysis. It suggested that PCDMs were closely related with disruption of erythroid cells in dyserythropoiesis of CDA II.

## 5. CONCLUSION

PCDMs were transformed during erythropoiesis and played a direct rule in the disruption and dissolution of late erythroid cells, resulting in ineffective erythropoiesis in patients with CDA II.
